# Supplementary material for: Metformin Enhances TKI-Afatinib Cytotoxic Effect, Causing Downregulation of Glycolysis, Epithelial–Mesenchymal Transition, and EGFR-Signaling Pathway Activation in Lung Cancer Cells
Source: Pharmaceuticals (Basel). 2022 Mar 21;15(3):381. doi: 10.3390/ph15030381 (PMC8955777; doi:10.3390/ph15030381)
Supplement: Supplementary file 1 [file pharmaceuticals-15-00381-s001.zip › Supplemetary.pdf]

Supplementary Material

Supplementary Table S1. Combination index values.

Supplementary Figure S1. Effect of metformin-afatinib combined treatment on EGFR signaling pathway. Cells were seeded and treated for 72 hours with their respective metformin-afatinib concentrations. GAPDH was used as constitutive control, western blot images were analyzed by ImageJ (NIH) and represented as bars. Images are representative for three independent experiments and the results of area are presented as mean ± SD of these three independent experiments. Data were normalized regarding endogenous control and statistically analyzed by one-way ANOVA. *$P\leq 0.05$, **$P\leq 0.01$, *** $P\leq 0.001$, **** $P\leq 0.0001$

Supplementary Figure S2. Effect of metformin-afatinib combined treatment on glycolytic enzymes and proteins. Cells were seeded and treated for 72 hours with their respective metformin-afatinib concentrations. GAPDH was used as constitutive control, western blot images were analyzed by ImageJ (NIH) and represented as bars. Images are representative for three independent experiments and the results of area are presented as mean ± SD of these three independent experiments. Data were normalized regarding endogenous control and statistically analyzed by one-way ANOVA. *$P\leq 0.05$, **$P\leq 0.01$, *** $P\leq 0.001$, **** $P\leq 0.0001$

Supplementary Figure S3. Effect of metformin-afatinib combined treatment on OXPHOS subunits. Cells were seeded and treated for 72 hours with their respective metformin-afatinib concentrations. we evaluated expression changes by western blot. Images are representative of three independent western blot experiments GAPDH was used as constitutive control, western blot images were analyzed by ImageJ (NIH) and represented as bars. Images are representative for three independent experiments and the results of area are presented as mean ± SD of these three independent experiments. Data were normalized regarding endogenous control and statistically analyzed by one-way ANOVA. *$P\leq 0.05$, **$P\leq 0.01$, *** $P\leq 0.001$, **** $P\leq 0.0001$
