# Supplementary figures and images for: Metformin Enhances TKI-Afatinib Cytotoxic Effect, Causing Downregulation of Glycolysis, Epithelial–Mesenchymal Transition, and EGFR-Signaling Pathway Activation in Lung Cancer Cells
Source: Pharmaceuticals (Basel). 2022 Mar 21;15(3):381. doi: 10.3390/ph15030381 (PMC8955777; doi:10.3390/ph15030381)

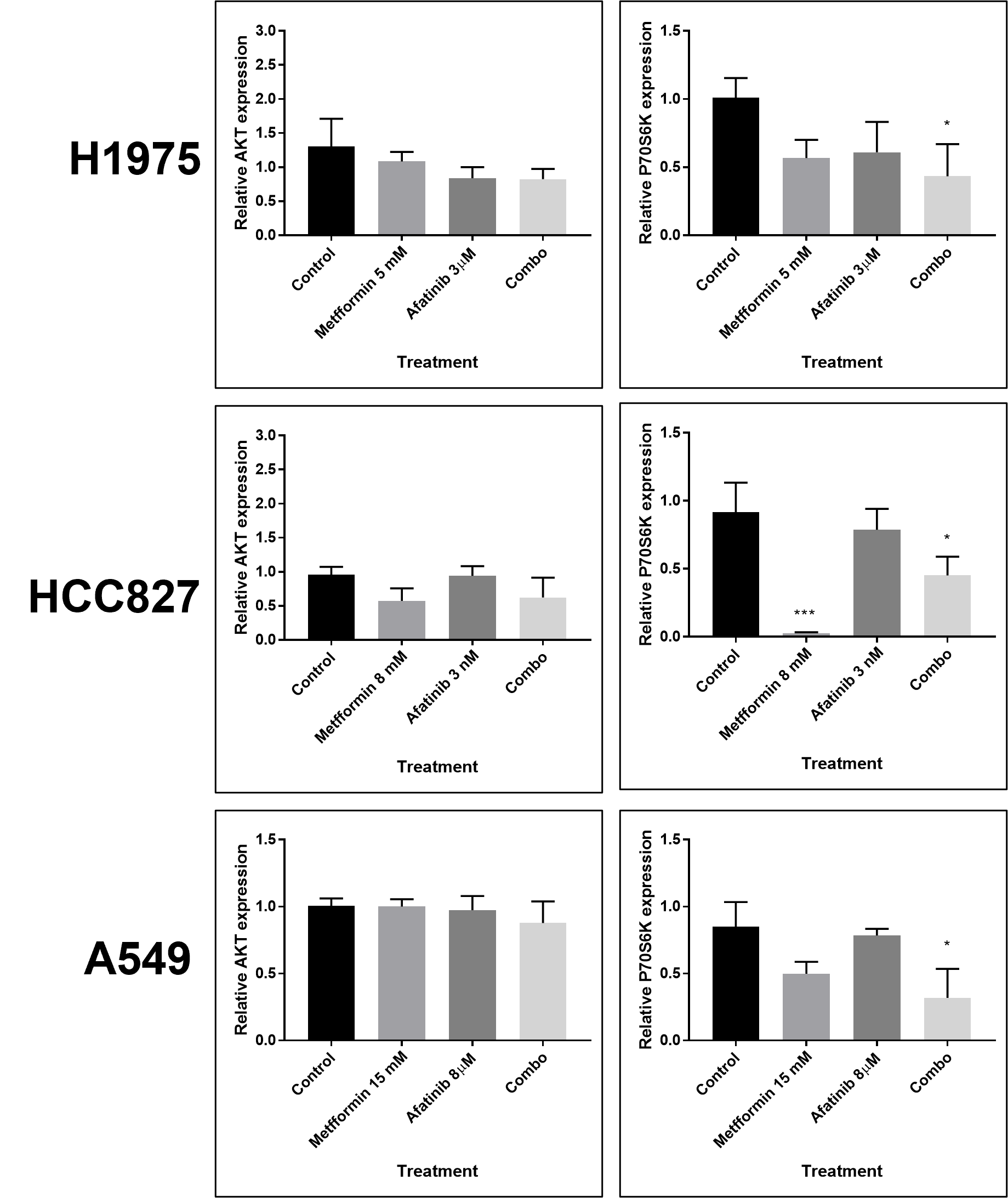

Supplement: Supplementary file 1 [file pharmaceuticals-15-00381-s001.zip › pharmaceuticals-1621776-Figure S1.png]

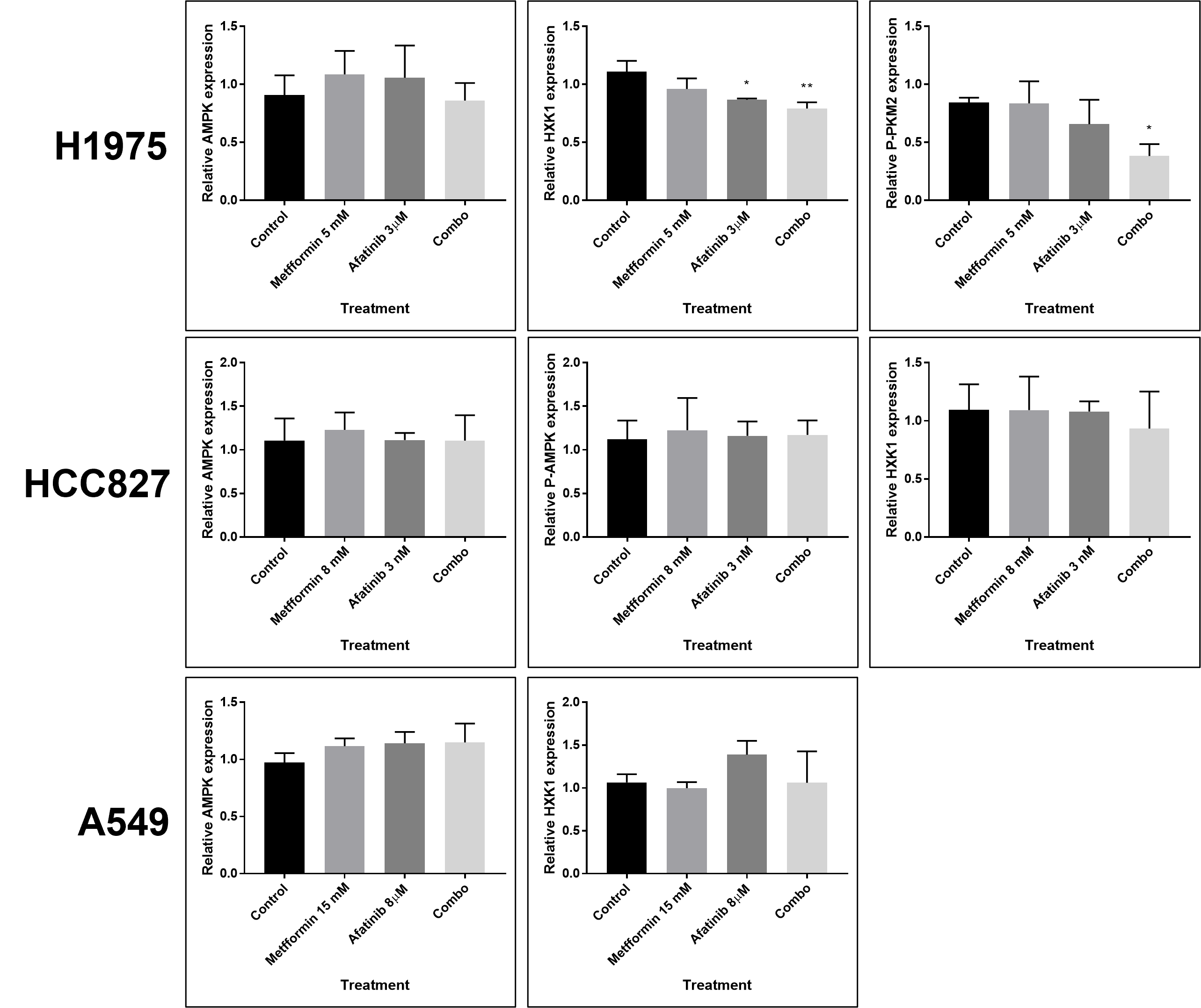

Supplement: Supplementary file 1 [file pharmaceuticals-15-00381-s001.zip › pharmaceuticals-1621776-Figure S2.png]

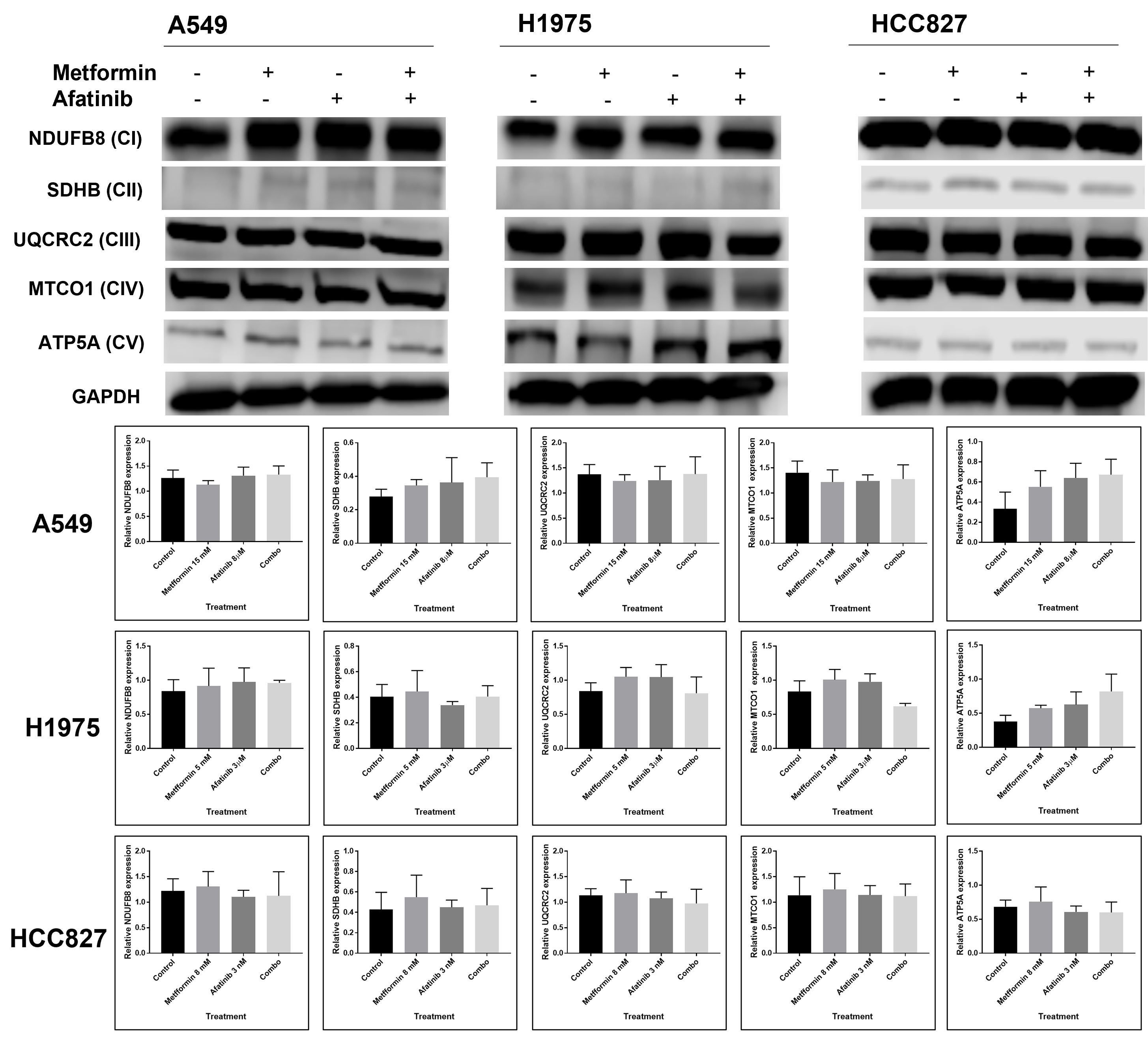

Supplement: Supplementary file 1 [file pharmaceuticals-15-00381-s001.zip › pharmaceuticals-1621776-Figure S3.png]
